# Theoretical basis for reducing time-lines to the determination of positive *Mycobacterium tuberculosis *cultures using thymidylate kinase (TMK) assays

**DOI:** 10.1186/1742-4682-6-4

**Published:** 2009-03-18

**Authors:** Misaki Wayengera

**Affiliations:** 1 Division of Molecular Pathology, Dept of Pathology, School of Biomedical Sciences, College of Health Sciences, Makerere University, PO Box 7072, Kampala, Uganda; 2 Restrizymes Biotherapeutics Uganda Limited, PO Box 16606, Kampala, Uganda; 3 School of Health Sciences, Kampala International University Western Campus, PO Box 71, Ishaka, Uganda

## Abstract

**Background:**

*In vitro *culture of pathogens on growth media forms a "pillar" for both infectious disease diagnosis and drug sensitivity profiling. Conventional cultures of *Mycobacterium tuberculosis *(M.*tb*) on Lowenstein Jensen (LJ) medium, however, take over two months to yield observable growth, thereby delaying diagnosis and appropriate intervention. Since DNA duplication during interphase precedes microbial division, "para-DNA synthesis assays" could be used to predict impending microbial growth. Mycobacterial thymidylate kinase (TMKmyc) is a phosphotransferase critical for the synthesis of the thymidine triphosphate precursor necessary for M.*tb *DNA synthesis. Assays based on high-affinity detection of secretory TMKmyc levels in culture using specific antibodies are considered. The aim of this study was to define algorithms for predicting positive TB cultures using antibody-based assays of TMKmyc levels *in vitro*.

**Methods and results:**

Systems and chemical biology were used to derive parallel correlation of "M.*tb *growth curves" with "TMKmyc curves" theoretically in four different scenarios, showing that changes in TMKmyc levels in culture would in each case be predictive of M.*tb *growth through a simple quadratic curvature, |tmk| = at^2^+ bt + c, consistent with the "S" pattern of microbial growth curves. Two drug resistance profiling scenarios are offered: isoniazid (INH) resistance and sensitivity. In the INH resistance scenario, it is shown that despite the presence of optimal doses of INH in LJ to stop M.*tb *proliferation, bacilli grow and the resulting phenotypic growth changes in colonies/units are predictable through the TMKmyc assay. According to our current model, the areas under TMKmyc curves (AUC, calculated as the integral ∫(at^2^+ bt + c)dt or ~1/3 at^3^+ 1/2 bt^2^+ct) could directly reveal the extent of prevailing drug resistance and thereby aid decisions about the usefulness of a resisted drug in devising "salvage combinations" within resource-limited settings, where second line TB chemotherapy options are limited.

**Conclusion:**

TMKmyc assays may be useful for reducing the time-lines to positive identification of *Mycobacterium tuberculosis *(M.*tb*) cultures, thereby accelerating disease diagnosis and drug resistance profiling. Incorporating "chemiluminiscent or fluorescent" strategies may enable "photo-detection of TMKmyc changes" and hence automation of the entire assay.

## Background

Infection with *Mycobacterium tuberculosis *(M.*tb*), the causative agent of tuberculosis (TB), is one of the leading global health challenges [1,2]. An estimated 8–10 million persons acquire tuberculosis annually, 2 million of whom die [1,2]. The global TB epidemic has been complicated by the human immunodeficiency virus (HIV) co-epidemic [3]. Interaction between HIV and TB: (i) is associated with a higher risk of progression to active *M*. *tuberculosis *infection (ATBI) among persons with existing latent infection (LTBI); (ii) leads to increased susceptibility to new infection with M.*tb*; (iii) renders diagnosis difficult and treatment/cure rates slow; (iv) results in a higher incidence of relapses; and (v) favors the evolution of drug resistance [1,4-6]. Amongst these, the emergence of drug resistance forms the deadliest challenge to controlling the TB epidemic. In HIV/TB high burden areas such as South Africa, drug resistance has extended from the first line anti-TB drugs to include the second line drugs spared for multi-drug resistant (MDR) TB, XDR [7]. Early detection of TB has a crucial role to play in controlling the epidemic here. In the past, diagnosis has been based on prediction of prior infection using the purified protein derivative (PPD)-tuberculin skin test (TST), detecting active TB by sputum smear staining using the Zeihl Neilson Stain (ZN) or culturing the organism on Lowenstein Jensen (LJ) medium; and radiographic imaging for TB-associated pathology [8,9]. More recently, newer molecular assays for TB have emerged based on serology (detecting the 38 kDa antigen, Early Secretory Antigen or ESAT-6, CFP10 and other secretory antigens), nucleic acid amplification (NAATS), phage amplification and line probe assays for MDR [10]. Despite the advent of these modern TB assays, largely because there are no inexpensive technology platforms exploiting related biomarkers, approaches based on *in vitro *cultures still form the most reliable and readily affordable method for diagnosing TB in many resource-limited settings [10]. In general, *in vitro *culture of pathogens on appropriate growth media forms a "pillar" for both infectious disease diagnosis and drug sensitivity profiling [9].

Conventional cultures of the slowly-growing tubercle bacilli (M.*tb*) on LJ medium, though highly sensitive for TB [10], are nevertheless time-consuming since they take about two months to yield observable growth, thereby delaying disease diagnosis and appropriate intervention. Although reading of culture results is still widely based on physical observation of the formation of bacterial colonies, modified assays that predict changes in '*in vitro *M.*tb *growth" by monitoring turbidity or metabolism of labeled metabolites are occasionally used to confirm the presence of actively growing tubercle bacilli [9-13] and to characterize drug resistance [9,14-16]. Specifically, some of these modified culture assays such as phage amplification assays, line probe assays and colorimetric redox-indicator methods can predict positive M.*tb *cultures (or drug resistance) in shorter times than the stipulated 2–3 months of culture on unmodified LJ medium. Most of these emerging TB assays are, however, still not readily available for routine use in most resource-limited settings where the TB burden is concurrently high [9]. We hypothesized that metabolomic-based assays of "para-DNA duplicative" changes during interphase could predict impending M.*tb *growth before actual growth occurs. Moreover, easier platforms such as chemilumniscence or fluorescence photodetection may be incorporated into these bioassays, thereby making them more affordable than the aforementioned existing methods for TB detection [10]. That hypothesis is derived from and based on several biological principles of the cell cycle, which are discussed below.

**First**, prokaryotic growth and proliferation, unlike that of eukaryotes, is predominantly achieved through division of single cells [17]. The period between the "birth" of two daughter cells from the parent cell and their own division is termed interphase. During interphase, cells undergo a resting (S) phase during which, though little physical activity is noted microscopically, DNA duplication occurs [17]. Since DNA synthesis precedes actual microbial division and proliferation, monitoring "DNA synthesis" may predict microbial growth changes well before the visible manifestation of actual microbial growth in culture.

**Second**, phosphorylation of deoxythymidine monophosphate (dTMP) is a critical step in the pathway leading to the synthesis of thymidylate triphosphate (TTP), a necessary precursor for DNA synthesis [18]. This process is catalyzed by enzymes called kinases or phosphotransferases [18]. Among mammalian cells, two phosphotransferases exist: thymidine kinases (types 1 and 2) and thymidylate kinase [18]. Sherley and Kelly have previously reported a positive correlation between levels of human TK and DNA duplicative changes in the cell cycle [19]. Within mycobacterial proteomes, however, no thymidine kinase has been detected [18]. Instead, mycobacterial thymidylate kinase (TMKmyc) carries out this critical phosphorylative function [18]. From this knowledge base, we proposed that levels of TMKmyc may similarly parallel DNA synthesis during the M.*tb *cell cycle [18,19]. We recently focused on research and development of an antibody-based biomarker for TMKmyc. To date, we have identified highly conserved surface linear peptides of TMKmyc as potential B-cell immunogens for *in situ *engineering of TMK-specific monoclonal antibodies (MAbs). Such TMKmyc-specific MAb(s) could be used to monitor of levels of TMKmyc in cultures of M.*tb*. In this paper, we attempt to provide a theoretical framework upon which we propose **reduction of time-lines for positive identification and drug resistance profiling among *Mycobacterium tuberculosis *(M. *tb*) cultures using thymidylate kinase (TMK) assays**.

## Methods and results

### 1. Arithmetical derivations of the systems biology of the tubercle bacillus growth curve

#### (a) Theoretical partition of the tubercle bacillus cell cycle

**Figure 1:**
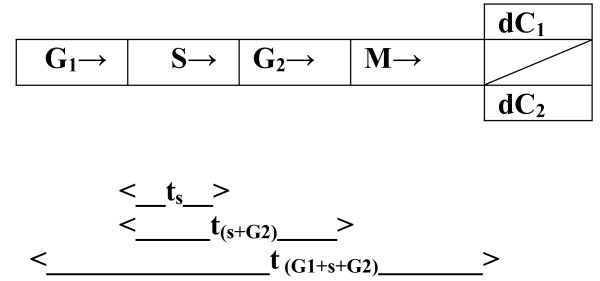
**The tubercle bacillus cell cycle**. The figure offers a graphic illustration of the cell cycle of the tubercle bacillus partitioned into the S (stationery or synthetic), G and M (mitotic) phases. Note that the times **t_**s**_**, **t**_**(s+G2) **_**and t**_**(G1+s+G2) **_are in a practical sense unknown.

We partitioned a "model" cell cycle of the tubercle bacillus, according each phase (G_1_, S, G_2 _and M) a time line [17]. These still ambiguous times have been designated: **t**_**s **_= time taken by the parent cell in the S phase; **t**_**(s+G2) **_= time taken for completion of a single 'cycle of the central dogma', i.e from DNA synthesis to protein expression; and **t**_**(G1+s+G2) **_= generation time of a tubercle bacillus (see Fig. 1). These are likely to vary from one cell type to another.

#### (b) Tubercle bacillus growth curve patterns and biometric algorithms

**Figure 2:**
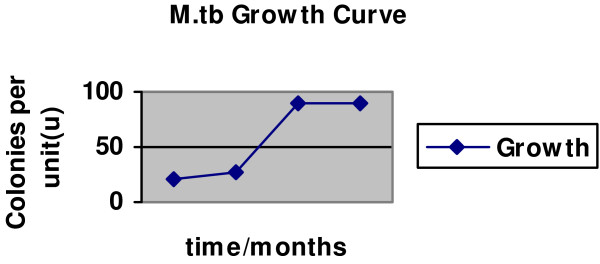
**A theorized tubercle bacillus growth curve (colonies per unit against time in months)**. The figure shows the typical S pattern characteristic of the growth of microbes in culture. Note the existence of an initial slow growth phase, followed by an exponential stage and finally a stationary phase.

As for all microbes, we assumed that the growth of tubercle bacilli follows the same "S" pattern, characterized by an initial "slow growth" phase, then an exponential phase and finally a stationary stage. The arithmetic of "S" curves is largely quadratic, the relationship between number of colonies per unit (μ) at any time (t) being stated as a derivative of the equation μ = at^2^+ bt + c (see Fig. 2). From this equation, we infer that the constants c, b and a respectively represent: c = the number of viable cells in the initial inoculum; b = the number of actively growing cells during the slow growth phase; and a = the cell division (or turnover) rate during exponential phase.

### 2. Geometric correlation of TMKmyc levels with the M.*tb *growth curve

We employed systems and chemical biology models to parallel "M.*tb *growth curves" theoretically with "TMKmyc curves" in four different scenarios. **First**, we assumed that the level of TMKmyc within an M.*tb *culture is equal to the total TMKmyc produced by all the functionally viable cellular units within all the colonies *in vitro*. This assumption was aimed at minimizing the impact of heterogeneity within the culture, which may contain an admixture (quasi) of dividing and non-dividing M.*tb*. Basically, as long as overall proliferation occurs, the cumulative levels of TMK would rise in proportion. **Second**, assuming that DNA synthesis occurs 2, 4, 6 and 8 days after inoculation (and that these are the equivalent time-lines for the G_1 _phase, t_G1_); then a further 58, 56, 54 and 52 days would be required for actual division of the tubercle bacilli to occur. Therefore, M.*tb *DNA synthesis may parallel the actual growth of tubercle bacilli but occur at a much earlier time. Hence the total secreted levels of enzymes such as TMKmyc synthesized during the cell cycle may parallel the "S" pattern of the microbial growth curve (and equally obey the quadratic equation); except that these changes are bound to occur much earlier.

**Figure 3:**
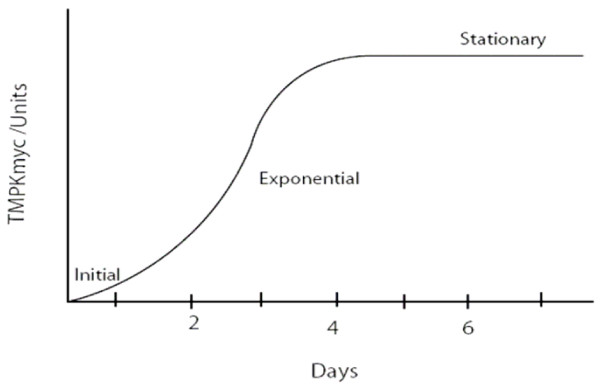
**Predicted pattern of TMKmyc variation in M.tb cultures**. This figure aims to illustrate the predicted pattern of variation in TMKmyc levels with the cell cycle, assuming that (1) levels of TMKmyc parallel growth changes and (2) DNA synthesis occurs 2–6 days after inoculation of the sample on LJ medium.

Overall, assuming that the cumulative levels of tmk in the culture is the total tmk derived from the individual cells in the culture, then, as for the microbial growth curves, it is highly probable that TMKmyc levels will obey an "S" pattern consistent with quadratic equations, but one that is manifest earlier. The levels of tmk per unit (|tmk|) at any time (t) on this gradient may therefore be represented as the product of the equation |tmk| = at^2^+ bt + c, where a, b and c are constants (possibly representing: a = multiple of the levels of TMPKmyc required to salvage a mole of dTMP during the exponential phase of M.*tb *growth; b = factor of levels of TMKmyc required to phosphorylate dTMP during the initial slow growth phase; and c = level of tmk required to phosphorylate a single dTMP molecule within the initial inoculum when no active division is ongoing) (see Fig. 3).

### 3. Prediction of drug sensitivity profiles based on TMKmyc levels rather than observation of M.*tb *growth changes (colonies per unit)

**Figure 4:**
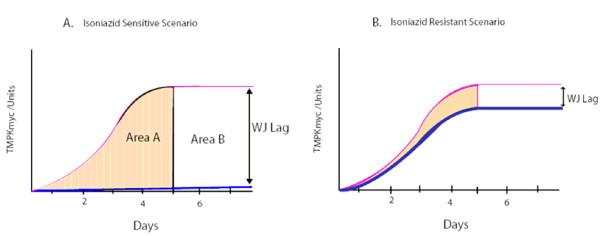
**Projected patterns of variation of TMKmyc curves in the drug sensitive and resistance scenario against a normal TMKmyc curve**. This figure shows theorized patterns of variation for TMKmyc curves in the drug sensitive (A) and resistance (B) scenarios against a background normal TMKmyc curve. Note that, from this illustration, one could say that the extent of resistance (or number of mutant phenotypes) present in scenario B is the difference between the areas under both curves shaded light yellow. Note also that for any drug resistance profiling based on tmk assays, resistance may be viewed as inversely correlated to TMKmyc levels, while sensitivity is directly proportional to levels of TMK. The difference between TMKmyc levels of test versus standard TMKmyc curve is denoted by a window "wj" or wayengera-joloba lag, the numerical value of which is "WJ". Overall, drug sensitivity ~WJ and resistance ~1/WJ.

Two drug resistance profiling scenarios were modelled, isoniazid (INH) resistance and sensitivity. In the INH resistance scenario, we projected that although the dose of INH in LJ is optimal for stopping M.*tb *proliferation, tubercle bacilli will continue to grow. Because the time-line for observing these growth changes (colonies/unit) is long, we believe that predictions of such growth changes based on TMKmyc assays (which parallel growth changes but occur earlier) may be a relatively quick way of determining drug resistance. In the INH sensitive scenario, since the drug inhibits or slows microbial growth, these changes may equally be predicted by TMKmyc levels recorded in the presence of the drug (growth is bound to be absent or at least much lower than in the absence of drug or in the drug resistance scenario) (see Fig. 4A and 4B for illustrations of INH sensitive and resistant scenarios respectively).

## Discussion

This paper provides the first ever theoretical and modeling framework to support the view that assays of enzymes involved in the synthesis of DNA precursors may be applied to shorten the time-lines to positive identification of microbial cultures (and possibly of other cell lines including cancers). Specifically: if variations in TMKmyc during the S phase of the tubercle bacillus cell cycle are independent of the time taken by the tubercle in other stages of the cycle, then TMKmyc assays are a potential predictive biomarker of *in vitro *M.*tb *growth that may be applied to reducing the time-lines to positive identification of *Mycobacterium tuberculosis *(M.*tb*) cultures. There are several existing methods for detecting TB within cultures, but they mostly lack inexpensive platforms for routine use within resource-limited settings where the TB burden is highest [10]. Moreover, many of these emerging TB assays [10] are not specific for detecting duplicative changes in M.*tb *DNA. We therefore felt it necessary to establish a highly specific M.*tb *DNA duplicative assay that may be mounted on inexpensive technology platforms that are currently widely used within resource-limited settings, such as lateral flow immunochromatography.

Overall, tmk-based assays are therefore likely to be cheap but still serve the purpose of accelerating diagnosis and drug resistance profiling of M.*tb *and possibly other infectious diseases in culture. **First**, we show here why variations in the durations of the G_1_, S and G_2 _phases of the M.*tb *cell cycle may account for a longer 'culture time' for M.*tb *(see Fig. 1 for illustration) than for other microbes. In principle, the generation time of any cell is determined by the time it spends in G_1 _and G_2 _[17]. Some cells, however, are known to go into deeper states of latency or dormancy (designated G_0 _or even G_00_), only reverting to the G_1 _stage when a need for reproduction, proliferation or regeneration arises [17]. Various environmental factors are known to initiate such deeper states of dormancy in microbes and eukaryotes alike including low temperature, absence of nutrients and low oxygenation states [20]; though the full range of factors that determine why tubercle bacilli go into related states of latency *in vivo *are yet to be fully established [1,21]. It is therefore unclear whether these deeper states of dormancy explain the slowly-growing nature of M.*tb *(especially since host immune factors also seem to play a significant role in arresting M.*tb *proliferation *in vivo *[21]). What is evident from our theoretical models, though, is that experiments aimed at establishing the actual values of the durations t_G1_, t_s _and t_G2 _for various microbes including M.*tb *may yield significant knowledge about the rate-limiting steps in their "growth" activity when cultured. For instance, using our proposed TMKmyc antigen capture assays, together with radio-labeled phosphorylated (dTMP^*r*^) nucleotide bases as metabolites, the time t_s _required for DNA duplication by the tubercle bacillus may be estimated.

**Second**, from the theoretical model that the timeline for the G_1 _phase of the M.*tb *cell cycle (t_G1_) is constant at 2, 4, 6 or 8 days, we inferred a parallel correlation of tubercle bacillus "growth changes" with TMK levels in each scenario (although changes in TMKmyc levels were predicted to occur much earlier, as noted in Figure 3B). These predictions are based on the hypothesis that since DNA synthesis precedes all cellular division, and TMKmyc is required to create the dTTP necessary for DNA synthesis, monitoring the levels of TMKmyc may predict actual growth changes well before such changes occur. Note, however, that the actual durations of the constituent phases of the M.*tb *cell cycle remain uncertain as stated above. One may therefore be justified in arguing that this presumption regarding the timing of the G_1 _phase of the M.*tb *cell cycle at days 2, 4,.... etc. is currently speculative, given that it is not yet clear which of the two stages, G_1 _or G_2_, constitutes the larger proportion of the generation time of the tubercle bacillus (see Fig. 2 and 3). Nevertheless, using this arbitrary presumption that DNA synthesis may occur only days after inoculation and that it is the G_2 _phase rather than G_1 _that accounts for much of the delay in physical manifestation of M.*tb *growth changes in culture, we show here that TMKmyc assays could predict TB growth *in vitro *as early as day 2 of culture. Moreover, we have successfully used it here to theorize that TMKmyc assays may also be exploited to distinguish drug-sensitive from – resistant forms of tuberculosis in culture (see Fig. 4). In this context, those test samples with evident rises in TMKmyc levels would in a practical sense be resistant to the drug being tested, whereas isolates where TMKmyc levels fail to rise are sensitive. **From the graphs 4 A and B**, one equally notes that: (1) tubercle bacillus drug sensitivity is an inverse factor of TMKmyc while existing resistance profiles are directly correlated to TMKmyc levels; (2) by measuring and quantifying the comparative difference between the areas under the TMKmyc curves (AUC) of resistant and sensitive isolates, it may be possible to quantify the existing levels of resistance (or number of resistant mutants). Assuming that the difference between TMKmyc levels in stationary phase between the tested M.*tb *isolates and the standard tmk curve is arbitrarily denoted a window "wj" of which the numerical value is "WJ"; then:

(1) Drug sensitivity ~WJ

(2) Drug Resistance ~1/WJ

Note that in equations I and II representing tmk variation in sensitive and resistant M.*tb *isolate scenarios respectively; the |tmk| levels within the test isolate culture are inversely correlated to the numerical value of the wayengera-joloba lag or "wj" window, also denoted "WJ".

The above Area Under Curve (AUC) method for drug resistance profiling offers a means of quantifying drug resistance. Such ability to quantify resistance is especially clinically significant within resource-limited settings where second-line TB chemotherapy options are limited and drugs with minimal evident resistance are useful in combination with other drugs to which the isolate being treated is not resistant. In other words, TMK assays would enable the derivation of "salvage" regimens from the limited available drug options. Supposing that tmk levels follow a quadratic curve as above, parallel to the "S" growth curve of microbes, then this AUC can be calculated as the integral (∫) of the equation: |tmk| = at^2^+ bt + c or simply 1/3 at^3^+1/2 bt^2^+ct. From this equation, it can also be derived that the drug sensitivity of tubercle bacilli is an inverse factor of the AUC while existing resistance profiles are directly correlated to the same Area. However, in order to compare the prevailing resistance profiles between two isolates, one would need first to define the time over which to make the above calculations (say t_1_-t_2_); although we recommend that future universal algorithms be based on the AUC between t = 0 and the t-value when the tmk levels first reach the stationery stage. Note also that TMKmyc assays have the advantage of being applicable to all drug scenarios without the need for modifications such as those required for colorimetric redox-indicator methods or nitrate reductase assays, which have been found to be highly sensitive only for rifampicin and isoniazid resistance testing [9]. Moreover, targeted detection of other related microbial enzymes such as HIV thymidine kinase may enable drug resistance profiling for HAART to be conducted within resource-limited settings, where inexpensive platforms for phenotyping or genotyping for drug sensitivity have remained mostly lacking [22]. In the case of the M.*tb *scenario, whether or not the proposed TMKmyc-based drug profiling assay would yield consistent results relative to several existing rapid drug resistance tests such as phage amplification, or line probe assays such as INNO-LipA Rif.TB(LiPA) or Genotype MTBDR, is a subject that requires future comparative evaluations once TMKmyc assays are in practical use [9].

Several limitations are evident in our theoretical model, which must be dealt with prior to the actualization of TMKmyc assays as a predictive biomarker for *in vitro *tubercle bacillus growth. **First**, it is currently unclear whether secreted TMKmyc is present in the culture medium at levels detectable by MAb(s). Specifically, existing data provide contradictory views about the possible secretory nature of TMKmyc. Munier-Lehmman et al. [18] have previously shown that TMKmt forms over 30% of all colony filtrate proteins in a strain of *E*. *coli *genetically engineered to express TMKmt. However, other studies on host sera using *in vivo*-induced antigen technology (IVIAT) [23-25] and crossed immunoelectrophoresis (CIE) [26], though identifying over 11 genes involved in M.*tb *metabolism, do not categorically list TMKmt among them. Note, however, that the absence to date of methods for specific monitoring of TMKmyc (such as the one we propose) among the latter studies [[23-25] and [26]] relative to work by Munier-Lehmann and colleagues [18] may possibly explain these discrepancies. **Second**, it is assumed that TB cultures constitute a homogenous mixture of actively proliferating cells. Within wild type M.*tb *cultures, however, this may not be the case as active and dormant tubercle may co-exist. Such heterogeneity of growth among the M.*tb *in culture may also affect TMKmyc secretory levels. We hold that since qualitative rather than quantitative measures of TMKmyc are required to determine M.*tb *culture positivity, TMKmyc-based assays are still valid for the purpose of reducing time-lines to the reading of TB cultures. What may be affected is drug resistance profiling, since this requires measurement of the levels of TMK in the culture. Therefore, for purposes of drug resistance profiling, more algorithms may need to be integrated into the TMKmyc assays to render them more standardized and reliable in the face of heterogeneity of M.*tb *proliferation *in vitro*. **Third**, in the absence of *in vitro *evidence to support the view that TMKmyc may be adequately and specifically detected by monoclonal antibodies, the proposed assays for TMKmyc levels in culture are still speculative. Whether TMKmyc-based assays will perform better than, worse than or the same as existing TB tests [10] therefore remains elusive and requires future comparative clinical trials. More work needs to be done to affirm the binding affinity and specificity of the antigen capture assays for TMKmt. This work is, however, complicated by the findings by Steingart and colleagues, who recently reported inconsistencies in most commercially available TB serodiagnostics [16]. **Fourth**, although it is clear that DNA duplication precedes actual microbial division [17], the duration of this process among various microbes including M.*tb *is largely unclear. Studies such as that by Sherley and Kelly [19] are therefore needed for M.*tb *and other infectious pathogens alike. Overall, if the above-listed potential shortcomings are overcome, the proposed antibody-based TMKmyc assays provide the flexibility to be mounted on a cheap technology platform for use within resource-limited settings. For instance, incorporation of "chemiluminiscent or fluorescent" strategies may enable "photo-detection of TMKmyc changes in culture" and thereby automation of the entire assay. Specifically, if the initiation of light emission is tailored to antigen (TMKmyc) capture, then chemiluminiscent or fluorescent TMKmyc-specific antibodies (CAbs and FAbs respectively) may be integrated within the LJ medium so that visual observation of color change or photometric measurements may be used to detect the levels of TMKmyc secreted. This simple automation could remove the need to conduct laborious quantitative ELISA or antigen capture assays to monitor TMKmyc levels, and thereby allow use by persons without extensive specialized training. Lastly, as TMKmyc is a specific antigen of M.*tb *[18], targeted detection of TMKmyc in sputum may provide an alternative strategy for diagnosing symptomatic TB to that based on staining for AFBs.

## Conclusion

TMKmyc assays may be applicable to reducing time-lines to determining positivity of *Mycobacterium tuberculosis *(M.tb). Moreover, TMKmyc assays may also serve to hasten drug resistance profiling *in vitro*. The inherent flexibility of the proposed antigen capture assay allows the potential incorporation of simple technologies such as chemiluminiscent or fluorescent strategies and could enable easy-to-use platforms to be built such as those based on "photo-detection of TMKmyc changes in culture", as well as automation of the entire assay.

## Competing interests

WM is affiliated to Restrizymes Biotherapeutics Uganda Limited. There are no potential sources of financial conflict of interest to declare.

## Authors' contributions

WM conceived the hypothesis behind this study, designed and undertook the theoretical and system modeling and wrote the final version of the manuscript.
